# ANTIGONE: A Programmable Energy-Efficient Current Digitizer for an ISFET Wearable Sweat Sensing System

**DOI:** 10.3390/s21062074

**Published:** 2021-03-16

**Authors:** Evgenia Voulgari, François Krummenacher, Maher Kayal

**Affiliations:** École Polytechnique Fédérale de Lausanne (EPFL), CH-1015 Lausanne, Switzerland; francois.krummenacher@epfl.ch (F.K.); maher.kayal@epfl.ch (M.K.)

**Keywords:** current-to-frequency conversion, current readout circuit, wearable electronics, low-current measurement, sweat biosensors, pH sensing, time multiplexing

## Abstract

This article describes the design and the characterization of the ANTIGONE (ANalog To dIGital cONvErter) ASIC (Application Specific Integrated Circuit) built in AMS 0.35 m technology for low dc-current sensing. This energy-efficient ASIC was specifically designed to interface with multiple Ion-Sensitive Field-Effect Transistors (ISFETs) and detect biomarkers like pH, ${\mathrm{Na}}^{+}$, $K^{+}$ and ${\mathrm{Ca}}^{2+}$ in human sweat. The ISFET-ASIC system can allow real-time noninvasive and continuous health monitoring. The ANTIGONE ASIC architecture is based on the current-to-frequency converter through the charge balancing principle. The same front-end can digitize multiple currents produced by four sweat ISFET sensors in time multiplexing. The front-end demonstrates good linearity over a dynamic range that spans from 1 pA up to 500 nA. The consumed energy per conversion is less than 1 J. The chip is programmable and works in eight different modes of operation. The system uses a standard Serial Peripheral Interface (SPI) to configure, control and read the digitally converted sensor data. The chip is controlled by a portable device over Bluetooth Low Energy (BLE) through a Microcontroller Unit (MCU). The sweat sensing system is part of a bigger wearable platform that exploits the convergence of multiparameter biosensors and environmental sensors for personalized and preventive healthcare.

## 1. Introduction

In the era of the Internet-of-Things (IoT) there is an emerging trend to improve the daily healthcare of individuals by monitoring physiological information such as vital signals like heart rate, physical activity and body temperature. The addition of data related to noninvasive wearable electrochemical sensors that detect target analytes in saliva or sweat [1,2,3,4,5] can allow real-time and continuous health monitoring. Sweat that can be easily and noninvasively collected from the surface of the skin is a biofluid whose composition analysis [6,7,8] can potentially lead to early diagnosis [9] of electrolyte imbalance or problems like hyponatremia or hypokalemia [10]. Biomarkers like pH, ${\mathrm{Na}}^{+}$, $K^{+}$ and ${\mathrm{Ca}}^{2+}$ detected in sweat allow the monitoring of hydration status and performance of athletes [11]. The sweat sensing wearable devices can continuously collect data without the need of laboratory equipment or hospital access [12].

Adding biochemical measurements in the existing electrophysiological fitness sensor platforms is critical since the combined data give a better insight in a wide range of conditions and diseases [13]. This work is part of a bigger platform that targets the convergence of all these health-related parameters in a wearable, miniaturized, wirelessly interconnected and energy-efficient system. The monitoring of heart rate in combination with body temperature, blood oxygenation and chemical biomarkers using a wearable sensing platform at home can improve everyday life and can support healthy ageing. The importance of personalized healthcare is evident in situations like the current COVID-19 pandemic.

Most sweat systems are targeting a single parameter [13,14]. However, there are some systems that read multiplexed combined data and wirelessly transmit them to a mobile device. A full system for multiplexed perspiration analysis is presented in [15] that measures glucose and lactate, ${\mathrm{Na}}^{+}$, $K^{+}$ levels and temperature. The front-end part is based on commercial off-the-shelf (COTS) transimpedance amplifiers (LT1462). In [16] a flexible microfluidic system that can simultaneously measure lactate, pH, sodium and temperature is reported. Another important system that is combining chemical (lactate) and electrophysiological data from electrocardiogram and is measuring concurrently is presented in [13].

The sensors used for sweat sensing should be capable of miniaturization and low power consumption. Ion-Sensitive Field-Effect Transistors (ISFETs) [17,18] can be fabricated in Complementary Metal Oxide Semiconductor (CMOS) technology and be used for ion detection [17,19,20]. For an ISFET sensor, the variation of the potential at the liquid and insulator interface is related to the threshold voltage. When the ISFET sensor operates in the subthreshold region, the variation of the drain current $I_{D}$ due to the reference electrode voltage change is defined as the readout sensitivity [21]. A multianalyte ISFET-based sensor able to detect pH, ${\mathrm{Na}}^{+}$, $K^{+}$ and ${\mathrm{Ca}}^{2+}$ in human sweat is presented in [22]. It has reported an ultra-low power consumption of 2 pW/sensor. The ISFETs that are used for tracking biomarkers in sweat have been characterized using an Agilent semiconductor parameter analyzer (HP4156) [23]. The analyzer applies the biasing voltages and measures the current. However, there is always the need of a miniaturized, energy-efficient, with good stability and sensitivity portable version of the full system that combines the ISFET, its control and the readout circuit. The ISFET-ASIC system can be integrated in a larger system that combines multiparameter biosensors and environmental sensors.

In this article we present a CMOS integrated, energy-efficient platform that can properly bias the ISFET sensors presented in [22] and digitize the multiple current signals produced by the different sensors in time-multiplexing. The novelty of the ANTIGONE ASIC compared to standard Analog-to-Digital Converter (ADC) and Digital-to-Analog Converter (DAC) blocks in any low-power MCU is related to the existence of more power management options and to the dedicated and versatile interface that better suits the ISFET’s operation.

The ANTIGONE ASIC can provide multiplexed measurements of multiple sensors using a single front-end with low power consumption in the direction of minimizing the power consumption of the whole platform. Its small size facilitates the connection of the ASIC to the sensors on a flexible surface. The ASIC is programmable to provide more flexibility, power down operation during the idle states and voltage adjustments for the biasing of the sensors. It also allows calibration and testing.

**Figure 1:**
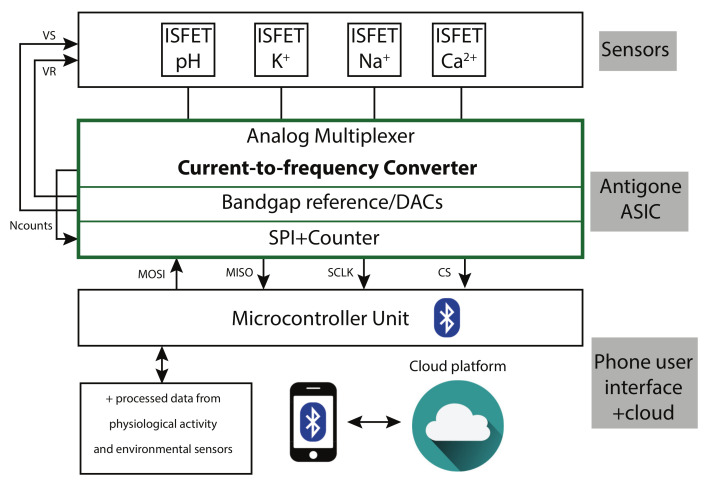
Wearable ISFET-based sweat sensing platform.

The block diagram of the wearable sensing platform is presented in Figure 1. The programmable ANTIGONE ASIC is the core of the system. It can measure the current produced by the ISFET sensors along with the temperature. The ASIC interfaces with a Microcontroller Unit (MCU) that controls the operation. The MCU processes the real-time data in addition to the data from the physiological activity and the environmental sensors that are uploaded to the cloud. A mobile application installed on a portable device communicates with the MCU over Bluetooth Low-Energy (BLE) interface.

The ANTIGONE ASIC was designed on a standard 0.35 m technology and its maximum current consumption is 25 A. It is also noteworthy that the chip is versatile and can be used as a general-purpose analog-to-digital front-end for sensing dc currents in the range of 1 pA-500 nA for MOSFET characterization.

## 2. System Architecture

The purpose of the ANTIGONE front-end is to properly bias and measure the ISFET-based sweat sensors. Two different architecture approaches were initially considered since the ISFET operates similarly to a Metal-Oxide-Semiconductor Field-Effect Transistor (MOSFET). A change in the gate/reference electrode voltage $V_{RE}$ is changing the current flowing between the source (S) and the drain (D). The ISFET parameter that could be measured could be either the reference voltage $V_{RE}$ or the source current $I_{D}$ when the other parameter is fixed [24].

In the first case, the ISFET sensor can be biased at ultra-low current in the range of 100 pA to 10 nA so that it works in the weak inversion region. In that case, the sensor reference electrode voltage $V_{RE}$ can be converted using an incremental ADC [25,26]. The sensor voltage can be converted against a bandgap voltage reference or a scaled kT/q PTAT voltage.

In the second case the reference electrode voltage $V_{RE}$ and the source voltage $V_{S}$ can be properly set using two embedded DACs. The source-to-drain voltage $V_{DS}$ is internally set to 200 mV and the $V_{RE}$ and $V_{S}$ are selected as such that the ISFET operates in weak inversion [18,27] that offers low-power consumption and avoids problems like channel-length modulation [28]. The $I_{DS}$ current of the sensor can be measured using a current-sensing analog-to-digital conversion circuit.

The first sensing architecture was considered initially, but the second architecture of current sensing was finally preferred by the project sensor design team.

The easiest way to convert a current into voltage is a resistive feedback amplifier or the transimpedance amplifier [29]. However, this circuit has some limitations due to noise, area and dynamic range. This is the reason why the ANTIGONE ASIC was designed based on the asynchronous Current-to-Frequency Converter (CFC) architecture through charge balancing [30]. This approach has been adopted for the 12-bit ISFET current digitization circuit since it demonstrates very good linearity and a wide dynamic range. The same operating principle had been also used for a different application where a dc-current produced from a radiation sensor had to be measured over a wide dynamic range of nine decades and demonstrated excellent linearity [31].

**Figure 2:**
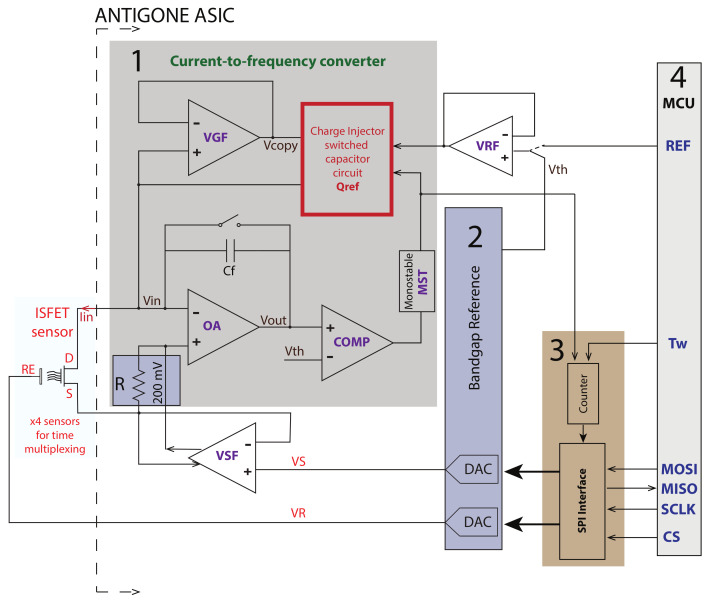
ANTIGONE ASIC Architecture. The main blocks are (1) the current-to-frequency converter, (2) the bandgap reference voltage, (3) the Serial Peripheral Interface (SPI) interface and the counter. The Microcontroller Unit (MCU) noted as (4) that is off-chip is the master/primary and controls the ASIC that is used in slave/secondary mode. On-chip analog multiplexers are connecting the input of the Current-to-Frequency Converter (CFC) to the ISFET sensors.

The architecture of the whole ISFET-ASIC system is presented in Figure 2. The different blocks that are shown with numbers from (1) to (4) are presented and discussed in the next subsections. An analog multiplexer is each time connecting the drain of each of the four external ISFET sensors to the input of the CFC that is marked as (1) for measuring. Additionally, there is the bandgap reference marked as (2) that generates the required voltages that are used in the circuit and the biasing currents for the amplifiers. The DACs that are biasing the reference electrode RE and the source *S* of the ISFET are resistive string dividers embedded in the bandgap circuit. The $V_{DS}$ voltage is designed to be 200 mV and is a scaled replica of the 2 V reference voltage.

Then, noted in Figure 2 as (3), there is a 16-bit Serial Peripheral Interface (SPI) that controls the ASIC operation and a 12-bit asynchronous counter. Finally, noted as (4) there is an external MCU used as the master/primary that controls the ANTIGONE ASIC.

### 2.1. Current-to-Frequency Converter

**Figure 3:**
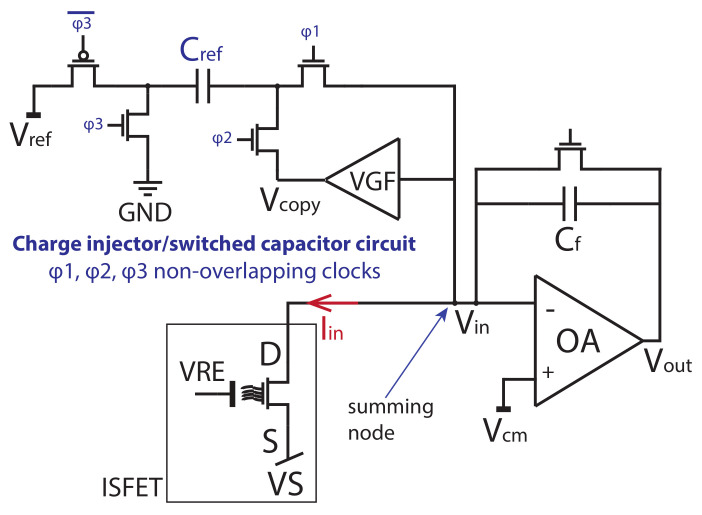
Integrator and switched capacitor circuit. The summing node of the currents is the $V_{in}$ node. Analog multiplexers connect each ISFET to the front-end.

The CFC that performs the analog-to-digital conversion consists of an integrator (OA), a comparator (COMP), a monostable (MST) and a charge injector $Q_{ref}$ circuit. The charge injector is implemented as a stray-insensitive switched capacitor circuit [32] as shown in Figure 3. The current $I_{DS}$ of the ISFET is integrated in the feedback capacitor $C_{f}$ of the integrator (OA). The output voltage $V_{out}$ increases until a defined voltage threshold $V_{th}$ is reached using the comparator. When the $V_{out}$ crosses that threshold voltage, a monostable (MST) is triggered that controls the nonoverlapping clock switches of the charge injector. The charge injector that is based on a switched capacitor circuit is injecting a constant charge $Q_{ref}$ into the summing node input $V_{in}$. That defined charge $Q_{ref}$ that is equal to $C_{ref}$$V_{ref}$, balances the charge that is stored in the feedback capacitor $C_{f}$ [31,33]. The charge $Q_{ref}$ has to be stable to guarantee a correct conversion. The $C_{ref}$ is the capacitor of the switched capacitor circuit. Using the charge balancing principle, the measured current is equal to the number of times $N_{counts}$ the charge $Q_{ref}$ was injected from the charge injector circuit into the input $V_{in}$ in a reference time window termed as $T_{w}$. This can be quantified using Equation (1).

(1) $$I_{DS}=\frac{N_{counts}Q_{ref}}{T_{w}}$$

**Figure 4:**
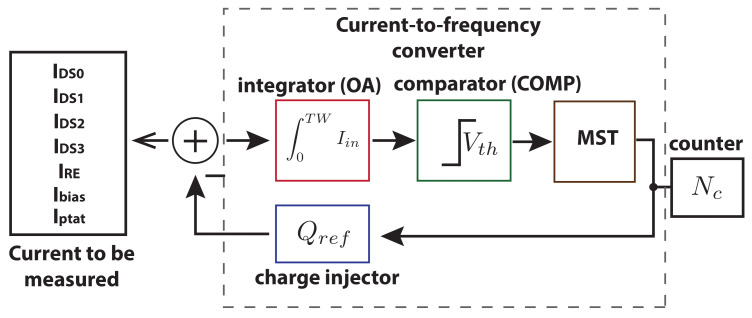
CFC and the different currents to be multiplexed.

The same current-to-frequency converter front-end is measuring four different ISFET sensors (pH, ${\mathrm{Na}}^{+}$, $K^{+}$ and ${\mathrm{Ca}}^{2+}$) in time multiplexing. The different currents ($I_{DS0}$-$I_{DS3}$) that are measured and the block diagram of the front-end are shown in Figure 4. Moreover, ANTIGONE ASIC can measure the reference electrode leakage current $I_{RE}$ to test the condition of each ISFET before performing a drain current measurement. A high leakage current (in the order of tenths of nA) shows that the ISFET is not working correctly. The reference current ($I_{bias}$) that is used to bias the amplifiers can be also tested for calibration purposes. Finally, the last current that can be measured is the $I_{ptat}$ so that the temperature can be also monitored. The simultaneous measurement of the temperature is important for the system calibration [17,34]. This way, the same low-power front-end can measure the multianalyte ISFET sensors, determine their state and also measure the reference currents for calibration without the use of extra circuitry.

### 2.2. Bandgap Reference Voltage and DACs

**Figure 5:**
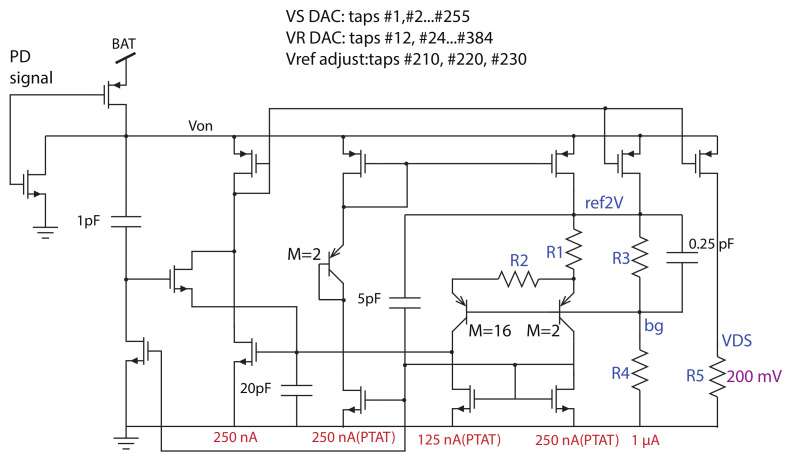
Bandgap voltage reference and resistive dividers.

In the CFC, the gain of the conversion is set by the product of the reference capacitor $C_{ref}$ of the switched capacitor circuit and the reference voltage $V_{ref}$. The $V_{ref}$ voltage has to be precise. A bandgap reference voltage was designed for this purpose and its microelectronic design is shown in Figure 5. The bandgap reference voltage was designed in order to generate the $V_{ref}$= 2 V voltage (noted as ref2V). That voltage is used as the switched capacitor’s reference voltage after buffering using VRF amplifier and as the comparator’s threshold voltage $V_{th}$. The same circuit is also used to supply the biasing currents of all the amplifiers used in the circuit and is extracted from the 250 nA PTAT.

The DACs that produce the voltages $V_{S}$ and $V_{R}$ that are biasing the ISFET sensors are embedded in the bandgap reference as resistive string dividers. The reference electrode $V_{RE}$ is biased using a 5-bit DAC that provides a voltage $V_{R}$ from 48 mV to 1536 mV in 32 steps of 48 mV. The source of the ISFET is biased using an 8-bit $V_{S}$ DAC. The buffered output of the DAC after the VSF amplifier has a voltage that ranges from 4 mV to 1024 mV in 256 steps of 4 mV each. The $V_{DS}$ voltage is equal to 200 mV and is generated by the current that passes through the resistor R5 of the bandgap. The noninverting input voltage of the integrator OA is equal to VS+200 mV due to the virtual ground.

All the analog blocks in this design, including the bandgap, are equipped with power down switches. This allows us to turn on each block separately in order to achieve a low-power consumption of the whole chip. The data acquisition is not continuous, so during the idle stages of the circuit the blocks are powered off. However, the bandgap is used to provide all the required voltages and currents and it has to be activated first. The power down (PD) signal that connects the $V_{on}$ either to the battery (BAT) through a PMOS transistor or to the ground through an NMOS transistor is also shown in this figure. Every block includes such a power down circuit.

### 2.3. SPI Interface and Counter

**Figure 6:**
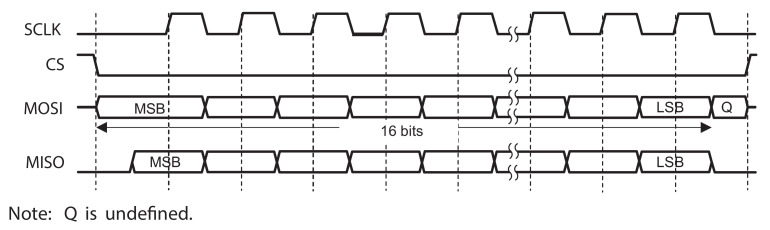
SPI transaction timing diagram and signals Serial Clock (SCKL), Chip Select (CS), Master Out Slave In (MOSI), and Master In Slave Out (MISO).

The ASIC communicates with the microcontroller with synchronous serial communication. The fully custom SPI interface of ANTIGONE consists of the main 16-bit register, a 12-bit asynchronous counter and eight 12-bit data registers that are responsible for the different operation modes of the ASIC. The ASIC is controlled through a 16-bit Serial Peripheral Interface (SPI) protocol using the pins MOSI, MISO, SCLK and CS. The timing diagram of an SPI transaction is shown in Figure 6. When the Chip Select (CS) is logic low, the SPI communication takes place and data is shifted in and out on the falling edge of the data clock signal. The digital input Master Out Slave In (MOSI) and the digital output Master In Slave Out (MISO) change their state at the falling edge of the Serial Clock (SCKL). The ASIC includes a Power-on-Reset (POR) circuit that initially sets the internal registers to a defined value.

The CFC operation and the measurement starts at the rising edge of an externally provided $T_{w}$ signal. The conversion finishes at the falling edge of the $T_{w}$ and the content of the counter is copied to the register and is acquired during the next transaction.

### 2.4. Microcontroller Unit (MCU)

The ASIC interfaces with the MCU that is used as the master/primary. The MCU drives the SPI interface and provides a clock frequency of 10 MHz. The first three bits of the SPI content are the control bits that are responsible for the different states of the ASIC operation. The MCU has the option to provide an external voltage reference REF = 2 V that can be also used for the $V_{ref}$ and $V_{th}$. However, this value is normally internally provided from the bandgap voltage reference circuit. The MCU is controlled from a mobile device over Bluetooth Low Energy (BLE) and a user-friendly app that can plot the collected data. For the first measurements and the initial testing of the platform, an Arduino Due was used. It is a microcontroller board based on the Atmel SAM3X8E ARM Cortex-M3 CPU. The ASIC that is presented in this article is part of a bigger platform and the MCU and BLE interfaces were selected from other partners that developed that part of the system.

## 3. System Design Requirements

The ASIC has to be supplied from a 3 V coin cell battery. The conversion time is in the range of 10 ms to 100 ms with a typical value of 30 ms. The current that is digitized spans from a few pA up to 500 nA. The lowest current that can be measured is limited by the leakage current. On the other hand, the front-end saturates when the charge produced by the charge injector is not enough to discharge the charge stored in the feedback capacitor. The maximum charge is equal to 1 pC ($Q_{ref}$ = 500 fF·2 V) and the maximum expected current of the ISFET is less than 500 nA. The front-end was designed with a low power consumption requirement. This is why the maximum current consumption when all the blocks are working is 25 A. The required energy per conversion is less than 1 J. The differential pair transistors of the amplifiers of the circuit were designed to work in the weak or moderate inversion region so that the transconductance is optimized for the same current investment. The current sources work in strong inversion for better matching. The integrated circuit has to be small in order to be connected to the ISFET chip. ANTIGONE ASIC’s size is 2 mm × 2 mm. Table 1 summarizes the system level requirements of the ASIC.

## 4. Programming of the ASIC and Different Operation Modes

The ANTIGONE ASIC can be programmed to work in different modes. The eight different modes of operation of the ANTIGONE ASIC are presented in Table 2. The first 3 bits of the SPI register are the control bits. Briefly, mode 1 sets the reading of the content of a specific register (R0-R7) and mode 2 corresponds to the selection of the I/O pads to be tested for the debugging of the system. We have the possibility to adjust and program some values using mode 3. These values include the trimming of the bias current IB of the different blocks using 2 bits and the tuning of the bandgap reference BG using 4 bits. The internal 2 V voltage, that is used for the comparator and the charge injector, can also be adjusted and calibrated. There is also the possibility to select between either an internal or an external voltage reference $V_{ref}$ that is provided from the MCU. Additionally, the reference capacitor value of the charge injector CR can be chosen between 50 fF or 500 fF according to the current that needs to be measured or the targeted acquisition frequency.

The monostable delay DL can also be trimmed using 2 bits. The monostable delay is important because it is related to the time allocated for the charging and discharging operation of the switched capacitor circuit. With a short delay, the charge cannot be formed properly and with a long delay there are frequency limitations in the maximum current. This can be explained by Equation (2). For a constant $Q_{ref}$, the charging and discharging time of the switched capacitor circuit that is equal to the monostable time MSO can affect the maximum measured current.

(2) $$I_{max}=\frac{Q_{ref}}{t_{charge}+t_{discharge}}=\frac{Q_{ref}}{2\;\cdot \;t_{MSO}}$$

Mode 4 operation takes care of the separate power supply of the analog blocks. They are activated separately and sequentially in order to contribute to the low power consumption. The activation timing depends on the time that is needed for the voltages to properly settle. Modes 5 and 6 are related to the programming of the $V_{S}$ and $V_{R}$ DACs. Modes 7 and 8 are responsible for the nodes that are biased and the nodes that are sensed. By sending two new mode 6 and 7 commands, a different ISFET sensor drain can be connected to the input, its $V_{S}$ node can be biased and its drain current be measured in time multiplexing by the CFC.

**Figure 7:**
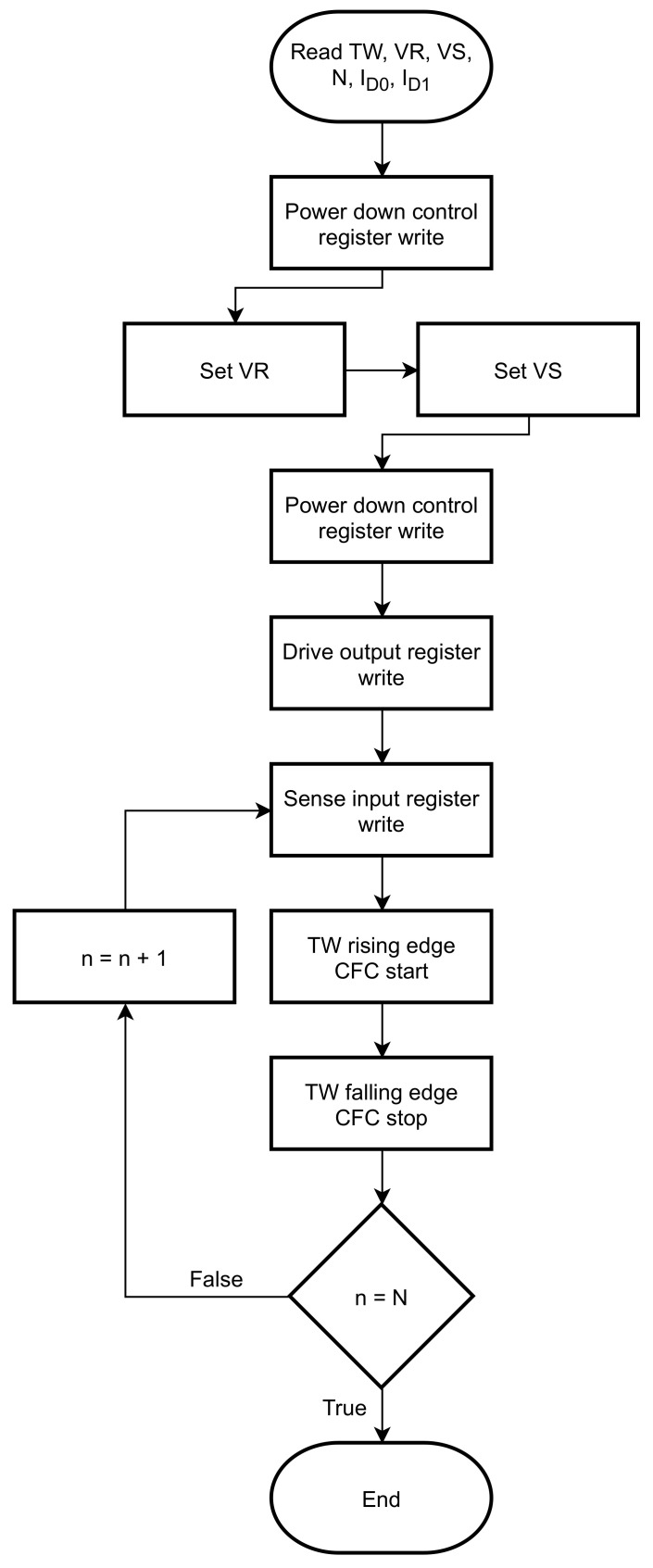
Flowchart of an example when measuring a current.

The flowchart in Figure 7 presents an example that corresponds to the measurement of current with the ASIC. The user can provide values such as the $T_{w}$, the $V_{R}$ and $V_{S}$, the number N of acquisitions for a current and the currents to be measured from the sensors, that in this case are $I_{D0}$ and $I_{D1}$. The power down control register command first activates the bandgap reference circuit and the VSF amplifier. Then, with modes 5 and 6 the $V_{S}$ and $V_{R}$ DACs are programmed. With a mode 4 command, after setting the biases, the analog blocks need to be powered and activated, in order to be ready to perform the analog to digital conversion. The ASIC is designed to be able to measure currents from three terminals of each of the four ISFET sensors. These three terminals are the drain, the source and the gate that can be set to “drive mode” or “sense mode”. For example, if $I_{D0}$ is measured, that means that $S_{0}$ and RE should be driven, so the corresponding bits ($S_{0}$ and RE) are set to “1” in the “drive mode” register. The “sense mode” bit that is active is the $D_{0}$. The conversion begins at the rising and stops at the falling edge of the $T_{w}$. Multiple measurements N can be performed for each current.

## 5. System Integration

**Figure 8:**
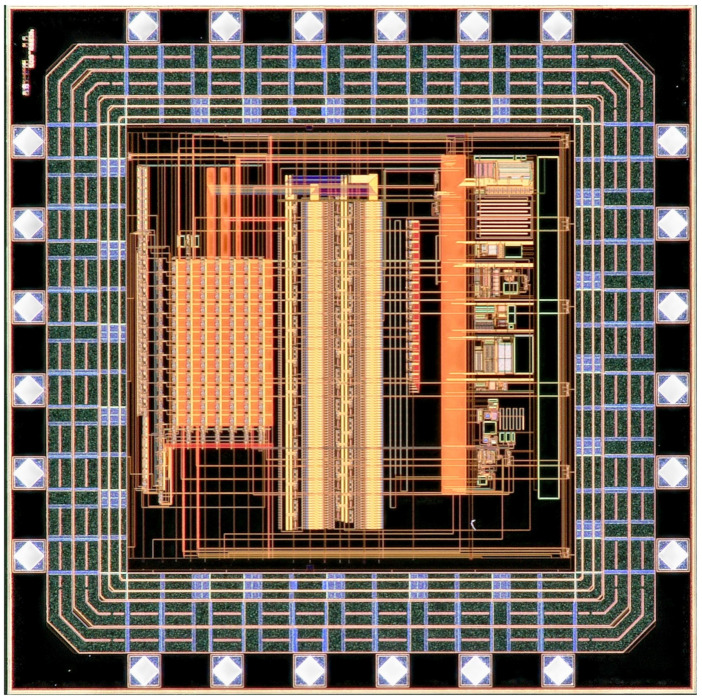
ANTIGONE ASIC microscopic photograph.

**Figure 9:**
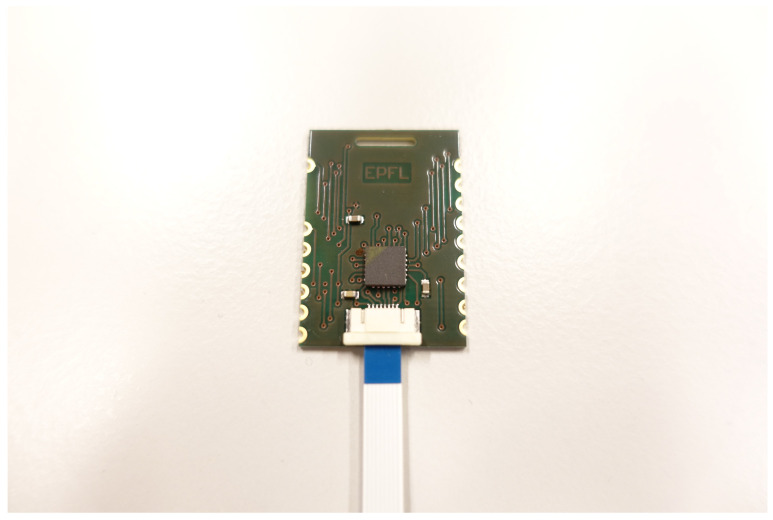
PCB with ANTIGONE packaged chip.

The ANTIGONE ASIC die is shown in Figure 8. It is fabricated in AMS 0.35 m technology, has 24 pads and its size is 2 mm × 2 mm. It was submitted through Europractice and it is encapsulated in a QFN 5 × 5 package. The chip was bonded on a simple PCB as shown in Figure 9 from one side and from the other side the ISFET ASIC was bondwired.

## 6. Results

The chip has been characterized for its electrical behavior and its functionality was verified. For the testing of the chip an Arduino Due was used. Firstly, the ASIC was calibrated using the configuration registers and mode 3. Then, the eight different modes of operation were tested separately and they were all functioning as expected. We could read back a register properly, power down each analog block separately, program the DACs and read out different sensors.

During a CFC conversion all blocks need to be powered. However, in order to achieve low-power consumption, some blocks can be turned-off during the idle states of the system. The activation order is noted with numbers from (1) to (6). Firstly the bandgap reference (REF) has to be turned on, then the DACs that are biasing the ISFET sensors and additionally the source voltage follower (VSF) amplifier. Then the blocks of the CFC that include the integrator (OA), the comparator (CO), the virtual ground buffer (VGF) and the monostable (MST) are activated sequentially. Table 3 presents the measured power consumption of each block after powering it up using the “Power write” configuration register. The measured values agree with the simulated values.

As an example of the calibration procedure, Table 4 shows the measured discharging pulse delay of the monostable according to the different capacitor that is connected using the two bits of DL[1:0]. The measured values are similar to the expected values after the post-layout simulation. The monostable provides the MSO pulse that is sent to the counter. However, the pulse duration is critical and has to be calibrated mostly for the maximum expected current case.

**Figure 10:**
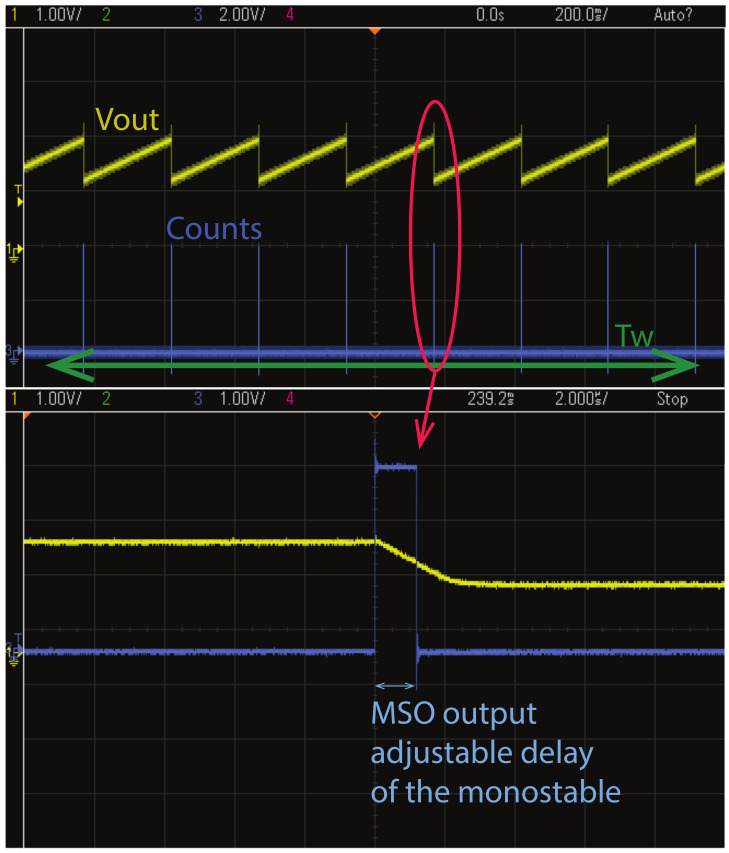
Analog output of the integrator $V_{out}$ and number of counts (MSO signal) shown in the oscilloscope. The CFC operating principle is shown.

The analog output of the integrator can be tested using an oscilloscope. I/O test pad register programming gives us the possibility to buffer a different analog output to a pad. The $V_{out}$ of the integrator (VOA in Table 2) is shown in Figure 10 with the yellow line. The digital output that is the MSO signal is also depicted in blue and its adjustable duration is measured. The principle of the CFC operation is shown in this figure where the drain-to-source current that is flowing in the ISFET is translated to the number of counts in the measuring time window $T_{w}$. The conversion takes place when the $T_{w}$ signal is HIGH and stops when it goes LOW. By counting the number of discharges, the input current is calculated.

**Figure 11:**
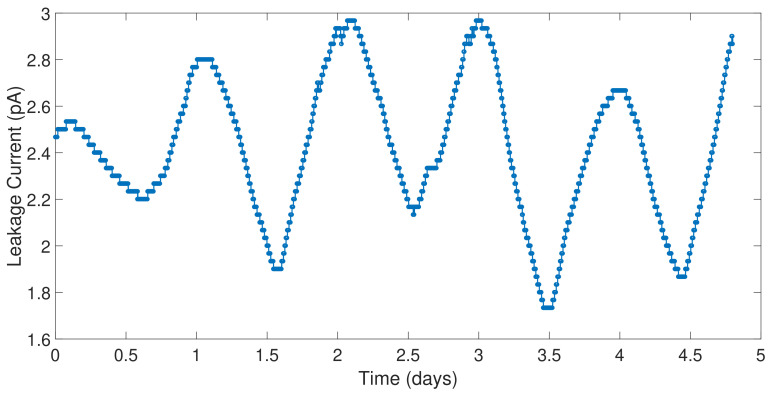
Leakage current measurement of one channel of the ANTIGONE ASIC.

The leakage current of the pads was measured in time multiplexing. It should be noted that leakage current in the order of some hundreds of femtoamperes can be tolerated since the minimum current $I_{DS}$ of the ISFET sensors was 100 pA. The net leakage current is related to the ESD protection, the switches of the analog multiplexer and the plastic QFN package [31]. However, the leakage current is dominated by the ESD protection leakage as shown in Figure 11 where it is affected by temperature. The plot depicts the leakage current of one pad during four consecutive days that is affected by temperature and by daycycle.

**Figure 12:**
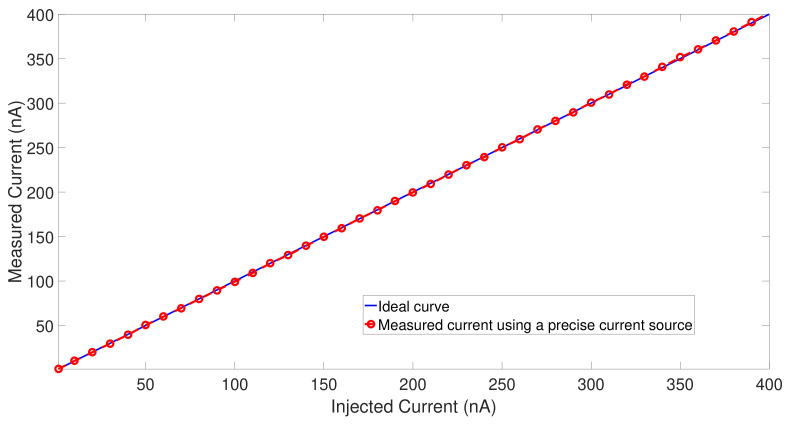
Linearity measurements from 1nA to 400nA using a precise laboratory current source from Keithley.

In order to test the linearity of the CFC, a laboratory current source (Keithley 263) was used to inject currents from 1 nA to 400 nA. The plot of the measured current versus the injected current from the current source is presented in Figure 12. The ANTIGONE ASIC demonstrates excellent linearity.

Measurements in a climatic chamber should be also performed in order to test the accuracy and the consumption of the front-end in temperatures from −40 °C to +85 °C. The user has to acquire multiple measurements of the ISFETs devices in order to have better statistics and noise averaging.

## 7. Discussion

**Figure 13:**
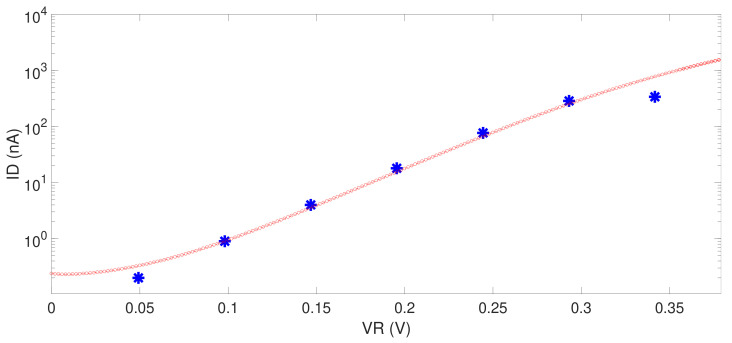
Comparison between the measurements acquired from a semiconductor parameter analyzer and ANTIGONE ASIC when the $V_{R}$ voltage is changing. The red points show the current measured with the parameter analyzer, and the blue points the current using the ANTIGONE ASIC.

The next step is to interface the ASIC with the ISFET sensor and acquire measurements using the whole system. Figure 13 shows the current $I_{D}$ (in logarithmic scale) versus the $V_{R}$ voltage. The current is acquired using a semiconductor parameter analyzer (in red) and ANTIGONE ASIC (in blue). In the case of the ASIC the $V_{R}$ was sweeping using the internal DAC. More measurements with the ISFET sensors have to be performed. At the time of the writing of this paper, we did not have access to the ISFET sensors.

Apart from this measurement where $V_{R}$ changes and the current $I_{DS}$ is measured, there is an alternative operation of the ASIC. The ANTIGONE ASIC can also work in a feedback loop. Since the embedded 8-bit and 5-bit DACs can be programmed by the MCU, the system can work in a way that the measured $I_{DS}$ current is always constant and the $V_{S}$ and $V_{RE}$ values are continuously adjusted to achieve that. So the MCU will have to continuously change the DAC values in order to keep a constant number of counts and a constant $I_{DS}$ current. In such a current-steering mode the $V_{RE}$ variations can provide a direct measure of the ISFET threshold shift due to the biomarker concentration.

## 8. Conclusions

This article presented the ANTIGONE ASIC that was designed to digitize the current produced by four ISFET sweat sensors. The front-end interfaces with the sensors and is also biasing them to proper voltages. The operating principle is based on the current-to-frequency converter through charge balancing in the input node. The ASIC has an internal bandgap voltage reference and can be programmed to perform eight different operations. The chip can be controlled by an MCU. It was designed for a noninvasive wearable system that measures biomarkers like pH, ${\mathrm{Na}}^{+}$, $K^{+}$ and ${\mathrm{Ca}}^{2+}$ in human sweat. The ASIC that interfaces with the ISFET sensors chip, has some important characteristics like a small size of 2 mm × 2 mm, low power consumption of 75 W, 12-bit resolution and programmability. The ultimate purpose of a programmable platform like this is the convergence of different sensors that lead to noninvasive assessment of vital signals and biomarkers that enhance wellbeing and preventive healthcare.

## Figures and Tables

**Table 1:** ANTIGONE ASIC specifications.

System Specification	Min	Typical	Max
Supply voltage	2.5 V	3 V	3.3 V
Conversion time window	10 ms	30 ms	100 ms
Energy per conversion			3 μJ
Supply current	10 μA	33 μA	100 μA
Operating temperature	10 °C		50 °C

**Table 2:** Operation modes.

Mode	Contr. Bits			Data Bits										
(1) Read register	0	0	0	X	X	X	X	R7	R6	R5	R4	R3	R2	R1	R0
(2) I/O test pad w	0	0	1	VR	VS	2V	BRE	INP	VGB	VBG	VOA	CO	MSO	MST	X
(3) Config reg. w	0	1	0	IB1	IB0	BG3	BG2	BG1	BG0	VR1	VR0	REF	CR	DL1	DL0
(4) Power w	1	1	0	X	X	X	X	X	MST	VGF	VRF	CO	OA	VSF	REF
(5) VR DAC w	1	0	0	X	X	X	X	X	VR4	VR3	VR2	VR1	VR0	X	X
(6) VS DAC w	1	0	1	X	X	X	X	VS7	VS6	VS5	VS4	VS3	VS2	VS1	VS0
(7) Drive out w	1	1	0	VS	VR	VD	RE	D3	S3	D2	S2	D1	S1	D0	S0
(8) Sense in w	1	1	1	X	Ibias	Iptat	RE	S3	D3	S2	D2	S1	D1	S0	D0

**Table 3:** Power activation matrix and power consumption of each block.

(1) Reference Voltage PTAT Current Source (REF)	(2) Operating ISFET Sensor Voltages (VSF)	CFC	
2.88 μA	2.38 μA + 3.85 μA	**(3) OA**	8.45 μA
		**(4) CO**	1.88 μA
		**(5) VGF**	4 μA
		**(6) MST**	0.89 μA

**Table 4:** Programming of the monostable delay.

Code DL	Capacitor Value	Pulse Delay (MSO)
00	250 fF	700 ns
01	375 fF	870 ns
10	500 fF	1 μs
11	625 fF	1.2 μs

## Data Availability

Data sharing not applicable.

## Abbreviations

The following abbreviations are used in this manuscript:

MOSFET	Metal Oxide Semiconductor Field Effect Transistor
CMOS	Complementary metal oxide semiconductor
ISFET	Ion-Sensing Field-Effect Transistor
ASIC	Application Specific Integrated Circuit
CFC	Current-to-Frequency Converter
ADC	Analog-to-Digital Converter
OA	Operational Amplifier
MCU	Microcontroller Unit
DAC	Digital-to-Analog Converter
SPI	Serial Peripheral Interface
POR	Power on Reset
MOSI	Master-Out Slave-Input
MISO	Master-Input Slave-Output
PTAT	Proportional to Absolute Temperature
